# Butyl Benzyl Phthalate in Urban Sewage by Magnetic-Based Immunoassay: Environmental Levels and Risk Assessment

**DOI:** 10.3390/bios12010045

**Published:** 2022-01-15

**Authors:** Xia Hong, Yin Cui, Ming Li, Yifan Xia, Daolin Du, Chengwu Yi

**Affiliations:** Institute of Environmental Health and Ecological Security, School of the Environment and Safety Engineering, Jiangsu University, Zhenjiang 212013, China; hongxia@stmail.ujs.edu.cn (X.H.); candyminicy@163.com (Y.C.); xiayifan@stmail.ujs.edu.cn (Y.X.); ddl@ujs.edu.cn (D.D.)

**Keywords:** butyl benzyl phthalate, contamination, magnetic-based immunoassay, risk assessment, urban sewage

## Abstract

A magnetic-based immunoassay (MBI) combined with biotin-streptavidin amplification was proposed for butyl benzyl phthalate (BBP) investigation and risk assessment. The values of LOD (limit of detection, IC_10_) and IC_50_ were 0.57 ng/mL and 119.61 ng/mL, with a detection range of 0.57–24,977.71 ng/mL for MBI. The specificity, accuracy and precision are well demonstrated. A total of 36 environmental water samples of urban sewage from Zhenjiang, China, were collected and assessed for BBP contamination. The results show that BBP-positive levels ranged from 2.47 to 89.21 ng/mL, with a positive rate of 77.8%. The health effects of BBP in the urban sewage were within a controllable range, and the ambient severity for health (ASI) was below 1.49. The highest value of AS for ecology (ASII) was 7.43, which indicates a potential harm to ecology. The entropy value of risk quotient was below 100, the highest being 59.47, which poses a low risk to the environment and ecology, indicating that there is a need to strengthen BBP controls. The non-carcinogenic risk of BBP exposure from drinking water was higher for females than that for males, and the non-carcinogenic risk from drinking-water and bathing pathways was negligible. This study could provide an alternative method for detecting BBP and essential information for controlling BBP contamination.

## 1. Introduction

As one important member of phthalic acid esters, butyl benzyl phthalate (BBP) is also widely used in plastics, clothing, automobiles, cosmetics and other industries [1,2,3]. Through its long-term use, BBP has been internationally released into water, soil, atmosphere and even organisms, causing high risks to environmental ecology and human health [4,5]. Many researchers have demonstrated that the ecological levels of BBP in water in China are relatively higher than those abroad [6,7]. In Kaveri River, India; False creek harbor, Canada; and Eleven Point River and White River, USA, the concentrations of BBP can reach a few dozen nanograms per milliliter [8]. The Yangtze River Delta region of China (e.g., Shanghai, Nanjing or Zhenjiang) is an area characterized by abundant water and a developed economy and has been shown to have a high-level of BBP contamination [9]. Environmental toxicology research has reported that the concentration values for 50% of maximal effect (EC_50_) for BBP are as follows: 0.51 mg/L in seabass, 0.55–0.66 mg/L in sole fish and 0.68–3.0 mg/L in small sheepshead fish [10]. Furthermore, the health and the ecological target values were set at 60 μg/L and 12 μg/L for BBP, respectively [11]. Improved determination of BBP environmental levels is of great significance for carrying out environmental risk assessments, protecting environmental and ecological security and safeguarding human health.

The current methods for detecting BBP include the following: high-performance liquid chromatography (HPLC) [12,13]; gas chromatography-mass spectrometry (GC-MS) [14]; high-performance liquid chromatography–tandem mass spectrometry (HPLC-MS/MS) [15]; and enzyme-linked immunosorbent assay (ELISA) [16]. Varieties of immunoassay have attracted the most attention for phthalic acid esters (PAEs) detection due to their cost-effectiveness and high-throughput screening capability [17], such as the ELISAs for dimethyl phthalate (DMP) [18]; for dibutyl phthalate (DEP) [19]; for di-(2-ethylhexyl) phthalate (DEHP) [20]; the fluorescence polarization immunoassay (FPIA) for dibutyl phthalate (DBP) [21];the polymerase chain reaction (PCR) immunoassay for DEP [22]; and electrochemical immunoassays for bis (2-methoxyethyl) phthalate (DMEP) [23] and DBP [24]. An ELISA method for BBP was reported in our previous study [16], but there have been no reports of other immunoassays for BBP. Moreover, whilst the investigation of environmental levels of BBP in urban sewage and its risk assessment are essential, these studies are currently inadequate.

Magnetic-particles have a significant advantage of rapid separation, purification and biocompatibility and have been widely used for analysis and detection [25,26]. The magnetic-based immunoassay (MBI) could combine the rapid separation ability of magnetic-particles and the obvious advantages of an immunoassay. The MBI method has gained widespread attention due to the benefits of its highly efficient magnetic separation and its simple detection procedure [27,28,29]. The biotin–streptavidin system is a very popular strategy utilized for improving signal intensity [30]. By using the biotin-streptavidin strategy, more labeled tracers could be combined with the reaction system to obtain a significantly enhanced signal. Thus, the developed MBI method combined with the biotin-streptavidin system could improve detection efficiency and sensitivity.

**Figure 1:**
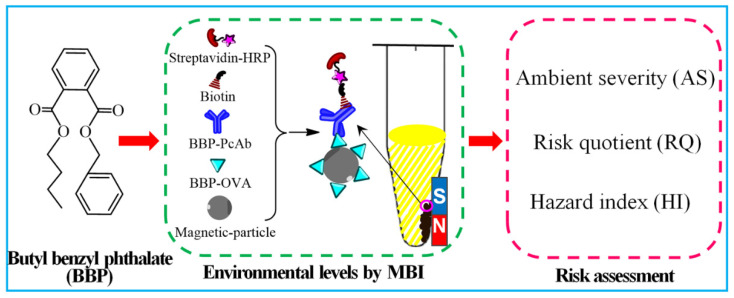
The schematic diagram of MBI method for the environmental levels and risk assessment of BBP.

Based on previous studies of antigens and antibodies for BBP, a sensitive and efficient MBI method of BBP detection was established (Figure 1) and then applied to evaluate BBP levels and to assess BBP risk in environmental water. Environmental water samples of urban sewage were collected from Zhenjiang, China, where water is suspected to be highly contaminated with BBP, mainly distributed in the urban sewage area. Three risk factors were assessed, including the values of ambient severity (AS), risk Quotient (RQ) and hazard index (HI). An alternative method for detecting BBP contamination and the environmental risk of BBP in the urban sewage area of Zhenjiang is presented in this study. This information will be beneficial to the early warning of BBP environmental risks and the protection of local environmental water and human health.

## 2. Materials and Methods

### 2.1. Reagents and Instruments

BBP and its analogue standards were purchased from Shanghai Yuanye Bio-Technology Co., Ltd. (Shanghai, China). Magnetic microspheres modified with carboxylic groups (5 mg/mL, 0.2 mm) were purchased from Suzhou VDO Biotech Co., Ltd. (Suzhou, China). Streptavidin-HRP, ovalbumin (OVA) and bovine serum albumin were procured from Solarbio Science & Technology Co., Ltd. (Beijing, China). Biotinyl-N-Hydroxy-succinimide (BNHS), sulfo-N-Hydroxy-succinimide (sulfo-NHS) and 1-[3-(Dimethylamino) propyl]-3-ethylcarbodiimide hydrochloride (EDC·HCl) were provided by Aladdin Reagent Co., Ltd. (Shanghai, China). Polyoxyethylene sorbitan monolaurate (Tween-20), 2-(4-Morpholino) ethanesulfonic acid (MES) and other reagents were purchased from Tansoole (Shanghai, China). Dialysis membranes (Regenerated cellulose, MWCO: 3500) were provided by Solarbio Science & Technology Co., Ltd. (Beijing, China). The anti-BBP polyclonal antibody (PcAb) and the coating antigen (BBP-OVA) were the same as in our previous published study [16]. The 0.05 mol/L MES buffer (pH 5.2), 0.05 mol/L carbonate-buffered saline buffer (CBS buffer, pH 9.6), 0.01 mol/L phosphate-buffered saline buffer (PBS buffer, pH 7.4), PBS buffer containing 0.05% Tween-20 (PBST buffer), PBS buffer containing 5% methanol, 0.5 mol/L Na^+^ (the working buffer) and citrate buffer containing 0.4 mmol/L 3′,5,5′-Tetramethyl benzidine and 3 mmol/L H_2_O_2_ (color-substrate buffer, pH 5.0) were prepared before use.

The absorbance at 450 nm was measured in an Infinite M1000 PRO reader (Tecan, Männedorf, Switzerland). A magnetic separation procedure was performed on a magnetic separator (Beisler, Tianjin, China). An ultrasonic processor KQ2200 was used for ultrasonic processing (Sumei, Suzhou, China). The microtiter plates (96-well) were used to carry out the MBI procedure (Corning, NY, USA). A Milli-Q purification system was used to prepare the ultrapure H_2_O (Merck Millipore, Darmstadt, Germany). An Agilent 1260 HPLC equipped with an ultraviolet detector (Agilent, Palo Alto, CA, USA) was used to validate the proposed immunoassay.

### 2.2. Preparation of Probes

The biotinylated antibody probe and magnetic probe were prepared in order to develop the MBI method. The biotinylated antibody probe was prepared by using the carbodiimide method [31,32]. The anti-BBP PcAb (dialyzed for 4 h in 0.1 mol/L CBS buffer, pH 9.2) was mixed with BNHS (3.8 mg in 100 μL DMSO), then incubated at 25 °C for 6 h. After dialysis in 0.01 mol/L PBS buffer overnight at 4 °C, the prepared biotinylated antibody probe was stored in 0.01 mol/L PBS buffer containing 50% glycerol and 3% bovine serum albumin. The NHS/EDC activation method was used to prepare the magnetic probe by coupling the BBP-OVA antigen and magnetic microspheres [33]. A volume of 50 μL magnetic microspheres modified with carboxylic groups was activated with 19.8 mg sulfo-NHS and 10.2 mg EDC in 950 μL MES buffer. After incubation at 25 °C for 30 min, the reaction mixture was washed twice by using an MES buffer under the magnetic field. Then, 50 μL of 5.3 μg/mL BBP-OVA antigen was added to 950 μL of activated magnetic microspheres and stirred at 4 °C overnight. After washing and separation, the prepared magnetic probe was blocked with 2 % bovine serum albumin and stored in a PBS buffer.

### 2.3. Development and Evaluation of MBI

Procedures of MBI: The appropriate magnetic probe (50 μL/tube, in PBS), the serial concentration of BBP standard solutions (50 μL/tube, in PBS containing methanol) and the biotinylated antibody probe (100 μL/tube, in PBS) were added to a tube and incubated for 30 min at 37 °C. Then, the reaction mixture was washed twice with PBST buffer (250 μL/tube) under the magnetic field for 1.0 min. The appropriate streptavidin-HRP (100 μL/tube, in PBS) was added and incubated for 10 min at 37 °C. After washing, separation and addition of the color-substrate buffer, the absorbance value was measured. A volume of 150 μL reaction solution was added to the 96-well and read at a wavelength of 450 nm.

Optimization and standard curve: The dosages of biotinylated antibody probe, magnetic probe and streptavidin-HRP and the contents of methanol and concentration of Na^+^ and pH values were selected to improve the sensitivity of MBI. The standard curve was obtained by plotting the logarithm of the concentration of BBP (ng/mL) against percent-binding values (B/B_0_, %). The B/B_0_ (%) value means the absorbance value with analyte against the absorbance value without analyte. Optimization criteria were based on the lowest value of IC_50_ and the highest ratio of B_0_/IC_50_.

Evaluation of MBI: The values of half-maximal inhibition concentration (IC_50_), limit of detection (LOD, IC_10_, 10% inhibition concentration of BBP for MBI) and detection range (IC_10_–IC_90_) were used to evaluate the sensitivity of MBI. The cross-reactivities (CRs) of MBI toward the BBP analogue were used to evaluate specificity. The correlation between the results of BBP detection between the MBI and the reference HPLC was used to evaluate the accuracy of the developed immunoassay. For the HPLC method, the water sample (5 mL) was extracted with acetonitrile (2 × 20 mL) for 30 min under shaking conditions. The organic phase was concentrated and cleaned using a solid-phase extraction column (Cleanert PAS, 1000 mg/6 mL, Agela Technologies, Wilmington, DE, USA). Then, the eluted BBP was concentrated and dissolved with 2 mL methanol and measured [34]. The chromatographic column was an Eclipse XDB2-C18 column (5 μm, 250 mm × 4.6 mm). The injection volume was 20 μL. The mobile phase was acetonitrile and water (2:1, *v*/*v*), with aflow rate of 1.0 mL/min. The detection temperature was 25 °C. The detection wavelength was 226 nm. In addition, the matrix effect of samples on MBI was evaluated and reduced by using the dilution method.

### 2.4. Sample Collection, Pretreatment and Detection

In this study, Zhenjiang (Jiangsu Province, China) was selected as the area for sample collection. This is an important city in the Yangtze River Delta at the intersection of the Yangtze River and the Beijing-Hangzhou Grand Canal. Zhenjiang has a very water-rich environment with many rivers. Due to rapid socioeconomic development and lack of effective protections for the environment, Zhenjiang’s numerous water resources and rivers have encountered serious pollution. Given the dense population of Zhenjiang, water pollution poses a potential threat to environmental ecology and human health. In recent years, Zhenjiang has been actively carrying out comprehensive treatment of its water environment. Monitoring BBP environmental levels and its risk assessment are necessary and valuable processes. The distribution of sampling comprised the main river flowing through the main populated areas and chemical processing factory, where BBP release and accumulation are likely to be most severe. The sampling coordinates of environmental water samples in Zhenjiang are shown in Table S1. The 36 samples of environmental water collected were filtered and stored in brown glass bottles at 4 °C. Then, the samples were diluted to reduce the matrix effect by using a working buffer containing optimal methanol, and then it was assessed by using the MBI method. The environmental levels of BBP contamination were summed up and used for the follow-up assessment.

### 2.5. Risk Assessment of BBP Contamination

Based on the environmental levels of BBP, the environmental health risk assessment of BBP contamination in environmental water in Zhenjiang was conducted, based on the three important reference values of ambient severity (AS), risk Quotient (RQ) and hazard index (HI). The key calculation formulas and parameters are listed in Table 1. Due to the different multimedia environmental goal (MEG) values [8,9], AS included AS for health (ASI) and AS for ecology (ASII). The value of AS reflects the risk of BBP to human health and the ecological environment. The ecological risk caused by BBP contamination can also be evaluated by using the value of RQ. As a small molecular compound of BBP, the non-cancerous hazard indexes of BBP for drinking water (HI_drink_) and for bathing (HI_bath_) were used to evaluate the risk of harm posed to local people following long-term exposure to BBP-contaminated environmental water. The criteria and grade of risk assessment are also shown in Table 1. All of these reference values correlate positively with the environmental levels of BBP (environment concentration, EC), meaning that the environment health risk increases with an increase in environmental levels of BBP.

## 3. Results and Discussion

### 3.1. Sensitivity of MBI

**Figure 2:**
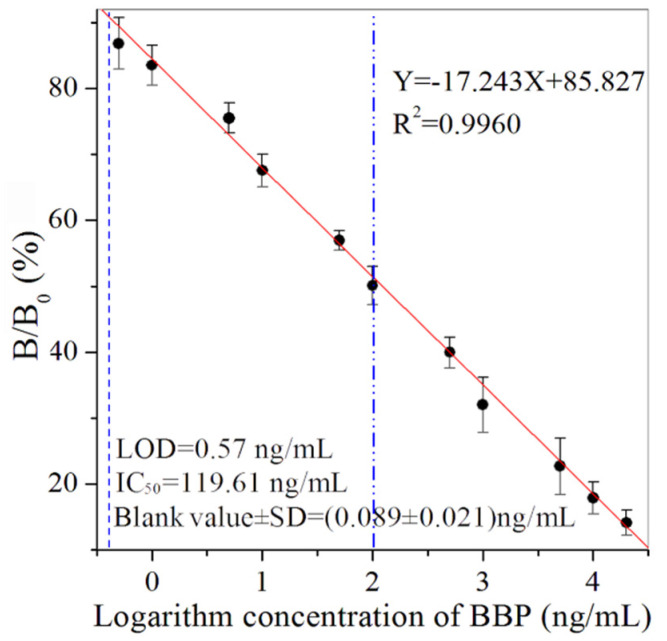
The standard curve of BBP by MBI (*n* = 3).

Under the criteria of the higher value of B_0_/IC_50_ and lower value of IC_50_, the dilution of the magnetic probe (0.6 ng/mL), biotinylated antibody probe (1.25 ng/mL) and streptavidin-HRP (18.9 ng/mL) were used as the optimal concentrations (Tables S2 and S3). In addition, the values of 5% methanol, 0.5 mol/L Na^+^ and pH 7.4 were selected as the optimal working buffer (Figure S1). According to the standard curve (Y = –17.243X + 85.827, R^2^ = 0.9960) (Figure 2), MBI had an LOD of 0.57 ng/mL, IC_50_ of 119.61 ng/mL and detection range of 0.57–24,977.71 ng/mL. The blank value of the MBI for BBP was 0.089 ng/mL, with a standard deviation (SD) value of 0.021 ng/mL. The sensitivity of the developed immunoassays could meet the requirements for the maximum limits of BBP [2,35]. The MBI for BBP showed advantages of simplifying the analytical procedure and shortening overall testing time. The sample pretreatment process was simple and the detection instrument was inexpensive.

The sensitivity of the proposed MBI could completely satisfy the requirement for detecting BBP. Compared with chromatographic methods, which need a complex sample pretreatment process and expensive instruments, MBI has the advantages of simplicity, low-cost and high-throughput screening of samples. The key properties of previous studies concerning BBP are listed in Table 2. The LOD of the ELISA method was 2.5 ng/mL, while the LOD of the MBI method could reach 0.57 ng/mL with a detection range of 0.57–24,977.71 ng/mL. The sensitivity of the MBI method of BBP detection shows improvements compared with previous ELISA methods, and the detection range is wider, allowing for improved detection incomplex environmental samples. Detection via the MBI method can be achieved by following three steps, within 40 min, while ELISA requires five steps and more than 2 h. The optimization results show that the MBI method used less antigens and antibodies, which could further decrease the cost of detection. Thus, the MBI method could shorten the overall testing time and simplify the analytical procedure and reduce cost.

### 3.2. Evaluation of MBI

CRs of less than 4.5% toward the BBP analogues indicate the high specificity of this alternative immunoassay (Table S4). When dilution multiples reached 1:8 (Figure S2), the matrix effects of environmental water samples could be minimized, and the detection of BBP by the MBI could be implemented exactly. The high accuracy of the MBI is demonstrated by good correlations between the results of the MBI and the results of reference HPLC (Y = 1.0262X + 0.027, R^2^ = 0.9786) (Figure S3). The results indicated that the proposed MBI could be simple, convenient, effective, specific and accurate for detecting BBP contamination.

### 3.3. Environmental Levels of BBP

A total of 36 environmental water samples were collected from the main rivers of Zhenjiang to investigate BBP contamination using MBI. As shown in Table 3, BBP contamination was found in 28 samples (77.8% of 36 samples), with environmental levels ranging from 2.47 to 89.21 ng/mL. BBP contamination was not detected in the remaining eight samples (22.2% of 36 samples). The average environmental level of BBP was 21.96 ng/mL, with the SD value ranging from 0.10 to 2.13 ng/mL. These data indicate that the accuracy and precision of the MBI were adequate for detecting BBP contamination in urban sewage samples. There were five samples with environmental levels of BBP above 50 ng/mL: S12 (84.11 ng/mL), S13 (89.21 ng/mL), S17 (57.06 ng/mL), S23 (71.02 ng/mL) and S24 (86.12 ng/mL). These samples with higher environmental levels of BBP were obtained from the middle and lower reaches of the Grand Canal. It was found that the river samples collected close to urban areas and densely populated areas were heavily contaminated with BBP. High environmental levels of BBP were also detected in samples collected close to commonly frequented areas and industrial sewage works. These significant environmental levels of BBP could be attributed to the widespread use and unceremonious discharge of BBP and the cumulative effect of BBP contamination. The results show that high environmental levels of BBP have become a commonly occurring problem in the main rivers in Zhenjiang. It should be noted that BBP levels were lower than the LOD value of the MBI method in samples S27, S35 and S36. The MBI determined these to be 3.96, 2.47 and 2.69 ng/mL, respectively. This study suggests that ecological environmental destruction and the potential health risks from environmental contaminants are closely related to human production activities and rapid economic development. Strict monitoring of environmental levels, assessment of environmental health risks and assessment of effective controls are recommended in order to protect ecological environmental safety and human health.

### 3.4. Comparison of BBP Contamination with Other Regions

As shown in Table 4, the environmental levels of BBP in environmental water samples from the other areas of China and from abroad were reviewed and compared with the results of this study. In China, the environmental levels of BBP from the urban river in Zhenjiang were similar to those in Huangpu River in Shanghai (9.411–86.395 ng/mL), lower than those in Xuanwu Lake in Nanjing (26.71–176.6 ng/mL) and higher than those in other researched water environments.

### 3.5. Risk Assessment

**Figure 3:**
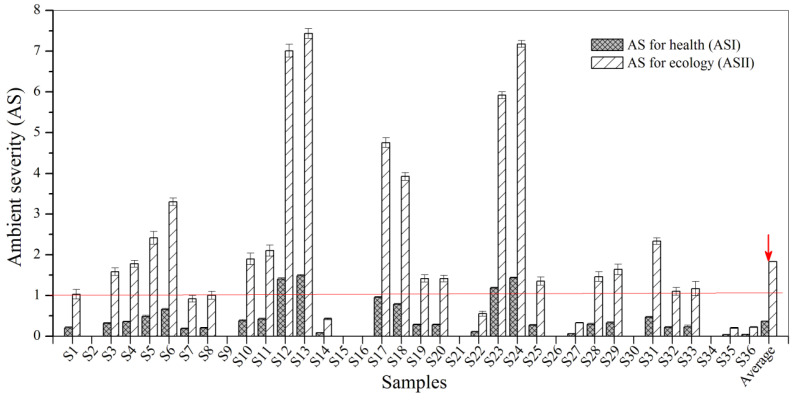
The AS for BBP in the urban sewage samples.

As shown in Figure 3, four samples (11% of 36 samples) had ASI values for BBP exceeding 1.0. These include the following: 1.40 for S12, 1.49 for S13, 1.18 for S23 and 1.44 for S24. According to the key criteria for the risk assessment of BBP (Table 1), most urban sewage samples indicate a negligible risk to health, whilst the above four samples indicate potential risk to health. It is worth noting that 61.0% of samples had ASII values greater than 1.0, and four samples had ASII values of more than 5.0. This indicates that the ecological risks of BBP in most sampling points were relatively large. Thus, BBP contamination in urban sewage samples from Zhenjiang poses a high potential risk to the ecosystem, and it is especially harmful to plants and animals in the water environment.

**Figure 4:**
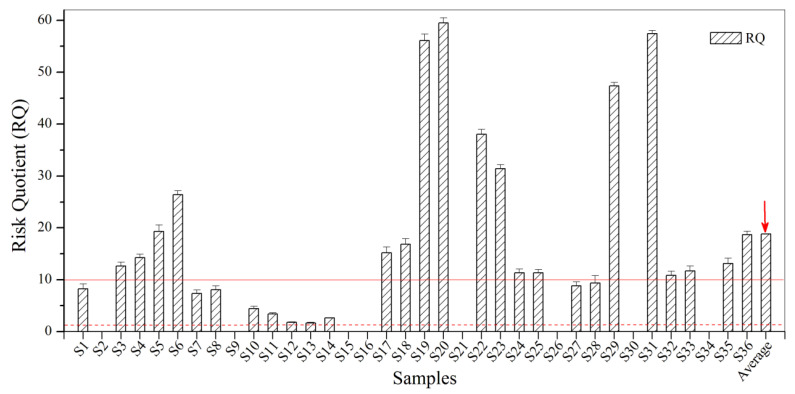
The RQ for BBP in the urban sewage samples.

According to the results presented in Figure 4, the values of RQ for BBP in the urban sewage samples were all between 1.0 and 100.0. Ten samples (27.8% of all samples) had RQ values lower than 10.0, which indicates a negligible risk or potential risk to the ecological environment. A total of 50% showed RQ values between 10.0 and 100.0, which indicates a low risk to the ecological environment. Taken together, BBP contamination of the ecological environment discovered in the urban sewage samples from Zhenjiang has the potential to be harmful to aquatic organisms and indicates a need for strengthened monitoring and management.

**Figure 5:**
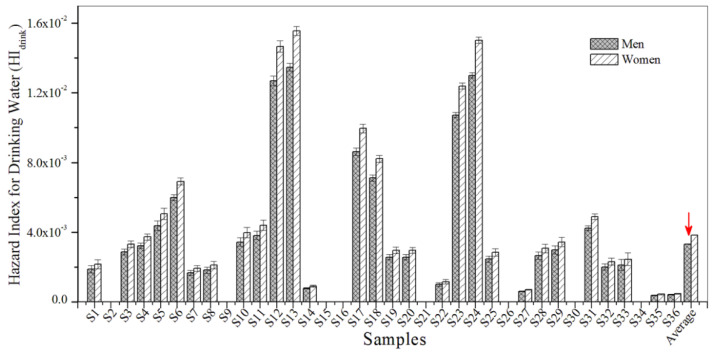
The HI_drink_ for BBP in the urban sewage samples.

**Figure 6:**
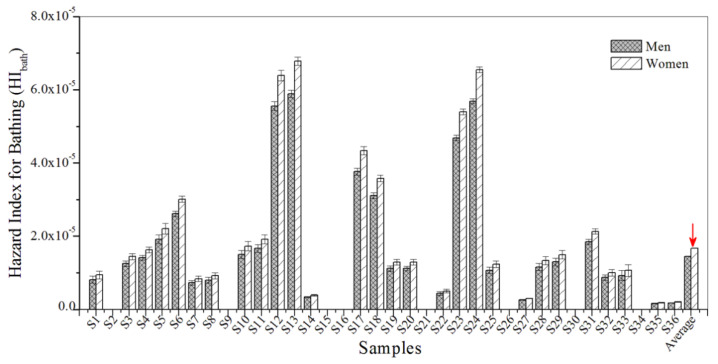
The HI_bath_ for BBP in the urban sewage samples.

A non-cancerous risk assessment was conducted in order to examine the hazard index for the exposure pathways of drinking water and bathing. Non-carcinogenic risk values for HI_drink_ and HI_bath_ were all lower than 1.0, indicating that non-cancerous risks were within the acceptable range and sufficiently low (Figure 5 and Figure 6). Furthermore, the results show that the values for HI_drink_ were generally higher than those for HI_bath_, which demonstrates that the non-carcinogenic risk of BBP contamination is slightly higher for women than for men.

Taken together, BBP contamination in the urban sewage samples from Zhenjiang has the potential to harm ecology, especially plants and animals. Currently, its risk to human health is limited. However, with the long-term exposure and bioaccumulation of the plasticizer, the risks to ecology and human health will rapidly increase. For the sake of environmental and ecological safety and human health, it is necessary to strengthen monitoring, comprehensive assessment and management of BBP contamination with respect to plasticizers.

## 4. Conclusions

A high-efficiency magnetic-based immunoassay has been developed and combined with the biotin-streptavidin system in order to detect BBP contamination in urban sewage from Zhenjiang, China. Compared with the previously reported ELISA, the developed MBI method offers a lower detection limit, a wider detection range and higher detection efficiency, with reduced utilization of antigens and antibodies. The average BBP-positive level of 21.96 ng/mL and the positive rate of 77.8% indicate that BBP contamination in this city is serious and widespread. In the risk assessment, environmental risks, ecological risks and health risks of BBP contamination were considered. The results show that BBP contamination risk in urban sewage from this region was generally lower, but it presents a higher risk towards ecology and might be harmful to animals and plants. The non-carcinogenic risk of BBP contamination through drinking water and bathing was within an acceptable range and sufficiently low. The risk assessment of BBP contamination in urban sewage is of great significance to the environment, ecology and human health. The environmental levels and risk assessment of BBP contamination in this study could provide a vital reference for assisting the local government in protecting the environment and ecology.

## Figures and Tables

**Table 1:** The key criteria and parameters for the risk assessment of BBP.

**Number**	**Equation**		
1	AS=EC/MEG		
2	RQ=EC/PNEC		
3	HI=CDI/RfD		
4	${\mathrm{CDI}}_{\mathrm{drink}}=\mathrm{EC}\times U\times \mathrm{EF}\times \mathrm{ED}/\left(\mathrm{BW}\times \mathrm{AT}\right)$		
5	${\mathrm{CDI}}_{\mathrm{bathe}}=\;I\times A_{\mathrm{sd}}\times \mathrm{EF}\times \mathrm{FE}\times \mathrm{ED}/(\mathrm{BW}\times \mathrm{AT}\times \mathrm{AR})$		
6	$I=2\times 10^{{}^{-3}}\times k\times \mathrm{EC}\times \sqrt{6\times \tau \times \mathrm{TE}/\pi }$		
**Parameter**	**Value**	**Parameter**	**Value**
MEG for health (μg/L)	60	MEG for ecology (μg/L)	12
Reference dose (RfD, mg/kg/day)	0.2	Average exposure time(AT, day)	10,950
Daily drinking water (U, L)	2	Body surface area (A _sd_, cm^2^)	16,600
Exposure frequency (EF, day/a)	365	Bath frequency (FE, time/day)	0.3
Exposure delay (ED, a)	30	Bath time (TE, h)	0.4
Body weight (BW_men_, kg)	66.2	Skin adsorption (k, cm/h)	0.001
Body weight (BW_women_, kg)	57.3	Delay time (τ, h)	1
Adsorption ratio (AR)	1	Adsorption dose (I, mg/cm^−2^/time)	Equation (6)
**Risk**	**Grade**	**Risk**	**Grade**
AS < 1	Negligible risk	RQ < 1	Negligible risk
AS > 1	Potential risk	1.0 ≤ RQ < 10	Potential risk
HI < 1	Negligible risk	10 ≤ RQ < 100	Low risk
HI > 1	Potential risk	RQ > 100	Seriously risk

EC, environmental concentration (environmental level of BBP); PNEC, predicted no-effect concentration (0.015 mg/L in this study); CDI, chronic daily intake.

**Table 2:** The comparison of results from two immunoassay methods.

Immunoassay	LOD (ng/mL)	Testing Range (ng/mL)	Testing Time (min)	Testing Steps	References
ELISA	2.5	2.5–1854.1	135	5	[16]
MBI	0.57	0.57–24977.71	40	3	This study

**Table 3:** The environmental levels of BBP in urban sewage samples.

Sample	Concentration ± SD (ng/mL)	Sample	Concentration ± SD (ng/mL)
S1	12.36 ± 1.43	S19	16.99 ± 1.08
S2	ND	S20	16.97 ± 0.97
S3	18.96 ± 1.11	S21	ND
S4	21.33 ± 1.02	S22	6.62 ± 0.71
S5	28.96 ± 1.89	S23	71.02 ± 1.02
S6	39.64 ± 1.12	S24	86.12 ± 0.99
S7	11.02 ± 0.98	S25	16.25 ± 1.21
S8	12.06 ± 1.17	S26	ND
S9	ND	S27	3.96 ± 0.10
S10	22.75 ± 1.69	S28	17.55 ± 1.41
S11	25.22 ± 1.63	S29	19.67 ± 1.57
S12	84.11 ± 1.89	S30	ND
S13	89.21 ± 1.48	S31	28.01 ± 0.97
S14	5.13 ± 0.28	S32	13.21 ± 1.16
S15	ND	S33	14.03 ± 2.13
S16	ND	S34	ND
S17	57.06 ± 1.43	S35	2.47 ± 0.19
S18	47.12 ± 1.15	S36	2.69 ± 0.13
Average	21.96		

ND, not detected. Each value represents the mean of three replicates.

**Table 4:** The review of BBP occurrence in environmental water samples.

Sampling Region	Sample	Range (ng/mL)	Ref.
Beijing, China	Plastic container	0.63–22.47	[36]
Wuhan, China	Yangtze River	1.14–1.23	[9]
Shanghai, China	Huangpu River	9.411–86.395	[6]
Beijing, China	Industrial and urban sewage	ND–62.5	[37]
Nanjing, China	Xuanwu lake	26.71–176.6	[38]
Shanxi, China	Fenhe River basin	ND–18.68	[39]
Karnataka, India	Kaveri River	ND–7.8	[7]
Mumbai, India	Trance Thane creek	2.5–20.5	[40]
Vancouver, Canada	False creek harbor	1.25–5.65	[41]
Ozark, USA	Eleven Point River and White River	ND–0.14	[8]
Zhenjiang, China	Urban sewage	ND–89.21	This study

ND, not detected.

## Supplementary Materials

The following supporting information can be downloaded at the following web site: www.mdpi.com/article/10.3390/bios12010045/s1, Figure S1: The Effect of methanol (A), Na+ (B) and pH value (C) on the sensitivity of MBI; Figure S2: The matrix effect of water samples on the sensitivity of MBI; Figure S3: The correlation between the MBI and HPLC for BBP; Table S1: Sampling coordinates of environmental water samples; Table S2: The key parameters for the proposed MBI; Table S3: The optimal concentration of antigen and antibody; Table S4: The results of cross-reactivity for MBI.
