# Supplementary material for: Butyl Benzyl Phthalate in Urban Sewage by Magnetic-Based Immunoassay: Environmental Levels and Risk Assessment
Source: Biosensors (Basel). 2022 Jan 15;12(1):45. doi: 10.3390/bios12010045 (PMC8773763; doi:10.3390/bios12010045)
Supplement: Supplementary file 1 [file biosensors-12-00045-s001.zip › biosensors-1539890-supplementary.pdf]

Supplementary data

Figure S1. The Effect of methanol, Na^+ and pH value on the sensitivity MBI.

Figure S2. The matrix effect of water samples on the sensitivity of MBI.

Figure S3. The correlation between the MBI and HPLC for BBP.

Table S1. The sampling coordinates of environmental water samples.

Table S2. The key parameters for the proposed MBI.

Table S3. The optimal concentration of antigen and antibody.

Table S4. The results of cross-reactivity for MBI.

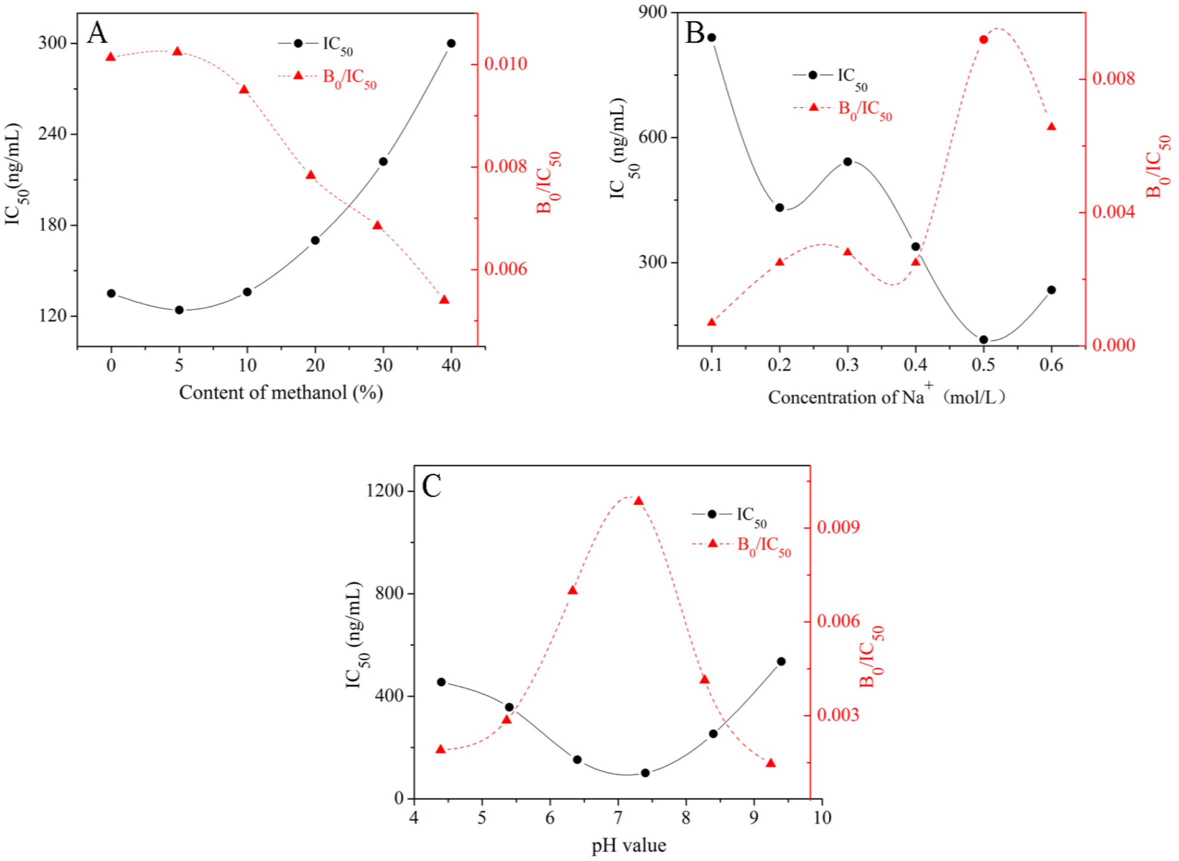

Figure S1. The Effect of methanol (A), Na^+ (B) and pH value (C) on the sensitivity of MBI.

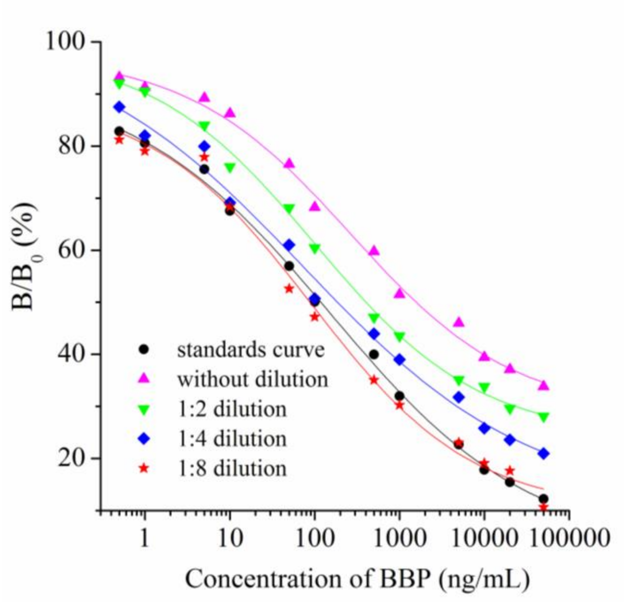

Figure S2. The matrix effect of water samples on the sensitivity of MBI.

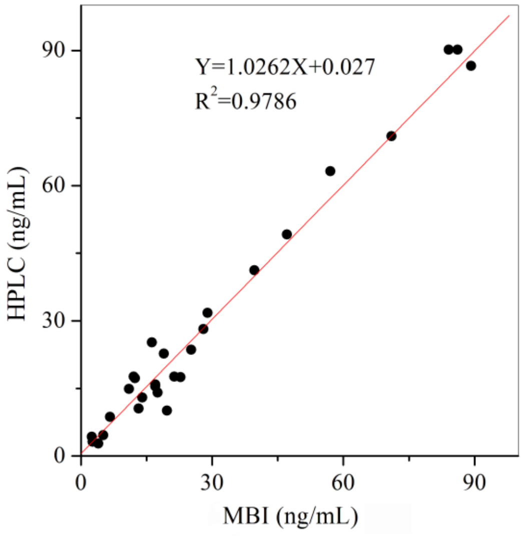

Figure S3. The correlation between the MBI and HPLC for BBP (The LOD was 0.15 ng/mL for HPLC).

Table S1. The sampling coordinates of environmental water samples.

Sample	Coordinates	Sample	Coordinates
S1	E119°22'21.02" N32°11'2.54"	S19	E119°30'13.39" N32°10'45.86"
S2	E119°23'27.70" N32°11'29.68"	S20	E119°29'57.81" N32°10'12.27"
S3	E119°24'7.87" N32°11'57.39"	S21	E119°29'45.45" N32°09'20.99"
S4	E119°24'12.82" N32°12'16.74"	S22	E119°30'14.64" N32°08'21.44"
S5	E119°25'12.18" N32°12'27.74"	S23	E119°30'24.51" N32°11'6.78"
S6	E119°25'2.28" N32°11'30.20"	S24	E119°31'14.84" N32°11'31.05"
S7	E119°25'46.88" N32°11'34.97"	S25	E119°13'27.51" N32°10'56.63"
S8	E119°24'53.63" N32°10'45.74"	S26	E119°32'45.34" N32°10'17.48"
S9	E119°24'54.25" N32°09'41.90"	S27	E119°33'29.33" N32°09'48.26"
S10	E119°26'20.57" N32°13'3.90"	S28	E119°33'16.35" N32°09'7.97"
S11	E119°12'41.93" N32°12'15.31"	S29	E119°33'4.61" N32°08'8.32"
S12	E119°26'29.62" N32°12'14.80"	S30	E119°33'23.15" N32°07'42.66"
S13	E119°27'29.05" N32°11'55.53"	S31	E119°34'0.94" N32°09'59.84"
S14	E119°27'44.57" N32°11'10.59"	S32	E119°34'48.67" N32°10'17.18"
S15	E119°27'40.84" N32°09'33.26"	S33	E119°35'10.30" N32°11'1.65"
S16	E119°27'32.09" N32°08'27.24"	S34	E119°41'51.12" N32°10'43.98"
S17	E119°27'55.71" N32°11'35.19"	S35	E119°42'18.26" N32°11'57.15"
S18	E119°29'3.90" N32°11'29.59"	S36	E119°40'33.35" N32°12'47.55"

Table S2. The key parameters for the proposed MBI.

Factor	Parameter	Factor	Parameter
Magnetic antigen probe	0.6 ng/mL	Methanol (*v/v*, %)	5
Biotinylated antibody probe	2.5 ng/mL	Na^+ (mol/L)	0.5
Streptavidin-HRP	18.9 ng/mL	pH value	7.4

Table S3. The optimal concentration of antigen and antibody.

B_0		BBP-OVA (μg/mL)								
		10.6	5.3	2.65	10.6	5.3	2.65	10.6	5.3	2.65
		Dilution ratio of magnetic antigen probe								
		1:2000			1:4000			1:8000		
Biotinylated antibody probe (ng/mL)	10	2.648	2.204	1.739	2.105	1.579	1. 421	1.789	1.378	1.012 69.8%[a]
	5	2.365	1.856	1.548	1.714	1.359	1. 256	1.556	1.126	0.894
	2.5	2.106	1.366	**1.056/ 65.4%** [a]	1.553	1.142	0. 884	1.341	0.879	0.578
	1.25	1.896	1.21	0.897	1.482	**1.005/ 70.3%**	0. 674	1.289	0.523	0.245
	0.625	1.483	1.136	0.699	1.189	0.752	0. 389	**1.045/ 64.8%**	0.312	0.098

[a] Inhibition: The 10 μg/mL BBP was used to test the inhibition experiment when the value of B_0 was approximately 1.0.

Table S4. The results of cross-reactivity for MBI.

Compound	Structure	IC_{50} (ng/mL)	CR (%)
Benzyl butyl phthalate (BBP)	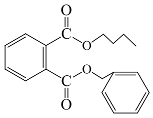	119.61	100
Dibutyl phthalate (DBP)	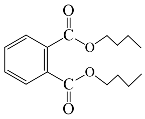	2682.93	4.5
Dimethyl phthalate (DMP)	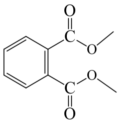	4514.03	2.7
Diethyl phthalate (DEP)	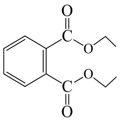	3781.25	3.2
Diisobutyl phthalate (DIBP)	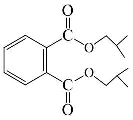	5873.79	2.0
Bis (2-ethyl hexyl) phthalate (DEHP)	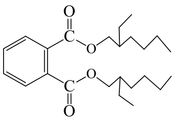	3878.21	3.1
Dicyclohexyl phthalate (DCHP)	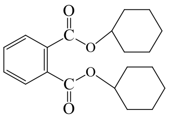	>10000	<1.2
Di-n-octyl phthalate (DnOP)	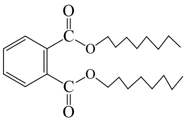	>10000	<1.2
Diisodecyl phthalate (DIDP)	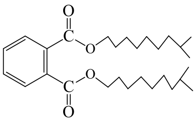	>10000	<1.2
